# Identifying anti-cancer drug response related genes using an integrative analysis of transcriptomic and genomic variations with cell line-based drug perturbations

**DOI:** 10.18632/oncotarget.7012

**Published:** 2016-01-25

**Authors:** Yi Sun, Wei Zhang, Yunqin Chen, Qin Ma, Jia Wei, Qi Liu

**Affiliations:** ^1^ Department of Central Laboratory, Shanghai Tenth People's Hospital, School of Life Sciences and Technology, Tongji University, Shanghai, China; ^2^ R & D Information, AstraZeneca, Shanghai, China; ^3^ Department of Plant Science, South Dakota State University, Brookings, SD, USA

**Keywords:** drug response, drug sensitivity, drug resistance, cancer cell line, personalized treatment

## Abstract

**Background:**

Clinical responses to anti-cancer therapies often only benefit a defined subset of patients. Predicting the best treatment strategy hinges on our ability to effectively translate genomic data into actionable information on drug responses.

**Results:**

To achieve this goal, we compiled a comprehensive collection of baseline cancer genome data and drug response information derived from a large panel of cancer cell lines. This data set was applied to identify the signature genes relevant to drug sensitivity and their resistance by integrating CNVs and the gene expression of cell lines with *in vitro* drug responses. We presented an efficient *in-silico* pipeline for integrating heterogeneous cell line data sources with the simultaneous modeling of drug response values across all the drugs and cell lines. Potential signature genes correlated with drug response (sensitive or resistant) in different cancer types were identified. Using signature genes, our collaborative filtering-based drug response prediction model outperformed the 44 algorithms submitted to the DREAM competition on breast cancer cells. The functions of the identified drug response related signature genes were carefully analyzed at the pathway level and the synthetic lethality level. Furthermore, we validated these signature genes by applying them to the classification of the different subtypes of the TCGA tumor samples, and further uncovered their *in vivo* implications using clinical patient data.

**Conclusions:**

Our work may have promise in translating genomic data into customized marker genes relevant to the response of specific drugs for a specific cancer type of individual patients.

## INTRODUCTION

Massive chemical compounds are currently being investigated for their potential use as anti-cancer drugs. Although a few of the compounds have been successfully used to treat defined patient subsets, a large set of the compounds is poorly characterized. It remains a great challenge to match the compounds with the subset of patients most likely to benefit from them. The ideal data set to achieve this goal would include the systematically characterized drug responses/sensitivities across a large cohort of patients. However, for most of the compounds tested, *in vitro* cell line systems provide the only available experimental data that can be used to identify predictive response signatures, and most of the compounds have not been tested in clinical trials. Comparisons have shown that cell lines mirror many aspects of tumor molecular pathobiology. Measurements of their genetic characteristics [1, 2] and therapeutic responses are well-suited for the development of strategies to identify the most predictive molecular signatures. For these reasons, several researchers have made efforts to characterize relationships between genomic profiles and drug responses [3–5], as well as to propose drug response prediction algorithms on the existing panel of cell lines [6–9].

Coupled with the accumulated *in vitro* cell line data for drug response identification, another issue that arises is drug resistance. It should be noted that a precise definition of “drug response” includes both “sensitive” and “resistant” response, where “sensitivity” refers to the effectiveness of different cell line responses to different drug perturbations, while “resistance” means the reduced effectiveness of a drug in the perturbation of a cell line. However previous literatures often mention “drug response” and “drug sensitivity” as two alternative claims of the same concept. Therefore in our study, for most cases readers can take “drug sensitivity” and “drug response” as identical terms. In addition, cancer drug resistance can be broadly divided into two categories, primary and acquired resistance [10, 11]. While primary drug resistance exists prior to any given treatment, acquired resistance occurs after the initial therapy. Understanding the mechanisms of drug resistance, especially primary resistance, is vital in the development of prospectively defined therapeutic sequences. Since the choice of first-line therapy determines second and subsequent line therapies, identification of the optimal first-line therapy is a priority for clinicians to develop efficient treatment strategies for patients. By pre-selecting those patients most likely to respond to drug treatment, clinicians can begin to optimize therapeutic strategies [12]. With the accumulated cell line data coupled together with their various genomic profiles and drug response data, *in vitro* cell line systems also provide us with an irresistible opportunity to uncover anti-cancer primary drug resistance mechanisms. Similar studies to this will provide useful insights for clinical trials if patient data are incorporated.

NCI-60 represents the pioneering cell line panel, where the responses of 60 genomically characterized cell lines have been measured for several thousands of compounds [13]. Recently, the Cancer Cell Line Encyclopedia (CCLE) cataloged genomic and drug response data for nearly 1,000 cancer cell lines [3]. Also the NIH launched the LINCS project, which aims to create a network-based understanding of biology by cataloging changes in gene expression and other cellular processes that occur when cells are exposed to a variety of perturbing agents [14]. As suggested in recent hallmark studies, screening very large cell line collections are expected to recapitulate known markers and identify novel molecular genomic determinants of drug response and drug resistance [4, 6]. The construction of a comprehensive dataset by integrating these valuable data sources may provide unprecedented power not only for drug sensitivity analysis but also for the discovery of drug resistance mechanisms. Nevertheless, a systematic screening for such markers using a comprehensive panel of *in vitro* cell line systems is still lacking. Furthermore, the implications of *in vitro* screening for the *in vivo* samples is also worthy of investigation.

In this study, we aimed to collect and curate comprehensive drug-cell line response data from various cell line data sources, and then based on this integrated dataset, we achieved the following goals: First, we designed a novel and efficient *in-silico* pipeline to identify signature genes that may correlate with drug response, especially primary drug resistance for different cancer types. We achieved this by integrating an analysis of transcriptional profiles with genomic characteristics, specifically the copy number variation of cell lines based on *in vitro* drug responses. Second, we presented a novel collaborative filtering-based drug sensitivity prediction model and measured it against the launched NCI-DREAM challenge on breast cancer cells by using the signature genes. Third, we conducted a comprehensive analysis of the identified signature genes related to drug resistance after excluding the cancer cell lines with disparate Copy Number Variations (CNVs) or mutation profiles. Fourth, we validated these signature genes and uncovered their implications *in vivo* using clinical patient data.

It should be noted that in our study we focused on the integration of the two cell line profiles, *i.e.,* the transcriptional profiles (the gene expression) and the CNV profiles. As suggested in two high impact studies on drug response analysis [4, 6], gene expression profiles were the most informative profiles for cell line characterization, therefore in our study this transcriptional feature was utilized as a preliminary profile for drug response analysis in different cell lines. In addition, previous studies have suggested that transcriptional changes corresponding to CNVs and alterations in gene dosage can be correlated with changes in expression levels [15, 16]. It is reported that in hematopoietic stem and progenitor cells, up to 28% of strain-dependent expression variation is associated with copy number variation, supporting the role of germline CNVs as key contributors to natural phenotypic variations in laboratory mice [17]. As indicated in the International HapMap project, CNVs capture 17.7% of the total detected genetic variations in gene expressions in 14,925 transcripts [18]. In a lung cancer study, approximately 78% of genes showed a positive correlation between CNV and gene expression levels [19]. Nevertheless, we found that a systematic investigation of drug perturbation integrated with genomic variation profiles is still lacking. Therefore, in our study we further combined gene expression profiles with CNV information to improve the identification of potential genes associated with drug responses. Our study excluded somatic mutation profiles, mainly due to the fact that: (1) The influence of somatic mutations on drug sensitivity are well understood [20–24]; (2) The mutation information for existing cell lines is very sparse, so it is not suitable for a comprehensive study in its current stage. (3) We already use the mutation information as a baseline for cell line description, thus making it possible to compare the cell line transcription and CNV profiles of the same mutation background (See Materials and Methods).

## RESULTS

### Overview of the *in-silico* pipeline to identify signature genes related to drug response

**Figure 1:**
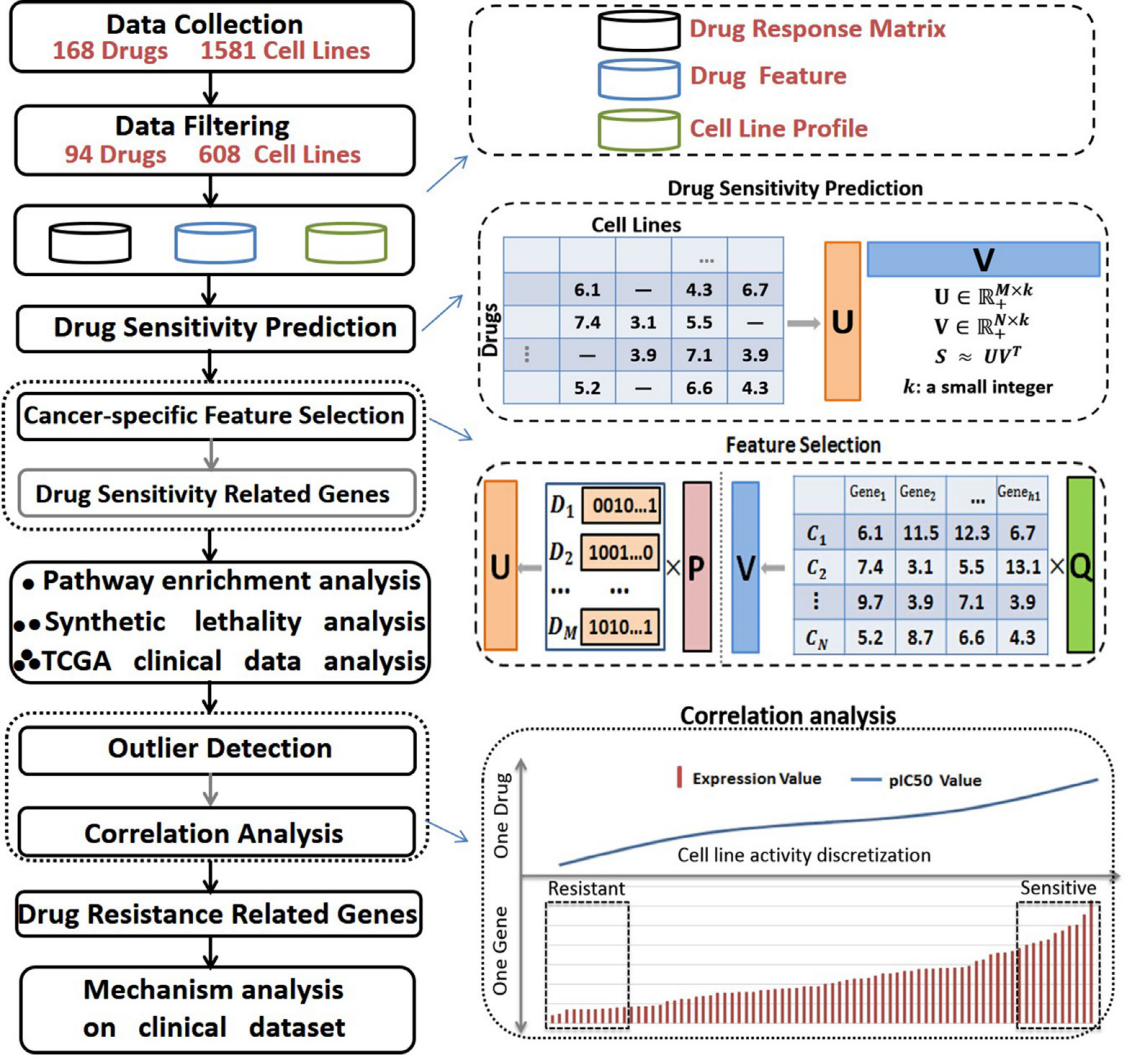
Standard pipeline Drug response information for 94 drugs on 608 cell lines was curated, and the baseline data for the cancer genome was carefully collected. A collaborative filtering based algorithm was applied to predict drug sensitivities in cancer cell lines, and group sparse lasso was applied to select signature genes of drug sensitivities for a specified cancer type. We conducted a comprehensive analysis of the identified signature genes through pathway enrichment analysis, synthetic lethality analysis, and validated these signature genes using TCGA clinical patient data. We further selected the signature genes that may correlate well to the primary resistance of a specific drug on a specific cancer cell type by incorporating CNV information with outlier detection and spearman correlation analyses.

A large compilation of baseline cancer genome data and drug response information derived from various cancer cell line data sources were used in our study to construct the curated dataset (See *Materials and Methods*). This dataset was applied to identify the signature genes relevant to drug sensitivity and to further their resistance by integrating CNVs and the gene expression of cell lines with *in vitro* drug responses. We designed an efficient *in-silico* pipeline to achieve this goal and identified the signature genes with simultaneous modeling of drug response values across all the drugs and cell lines. The pipeline efficiently integrates heterogeneous cell line data sources and has proven to be highly accurate in in predicting sensitivity (Figure 1, See Materials and Methods). Specifically, a collaborative filtering based algorithm [25] incorporating group lasso was introduced for drug sensitivity prediction and signature gene identification. These two methods are explained in detail in Additional file 1. Then for each drug, the signature genes that may correlate to the drug resistance of a specific cancer cell type were further screened (See Materials and Methods).

### Performance of the *in-silico* drug sensitivity prediction pipeline

**Figure 2:**
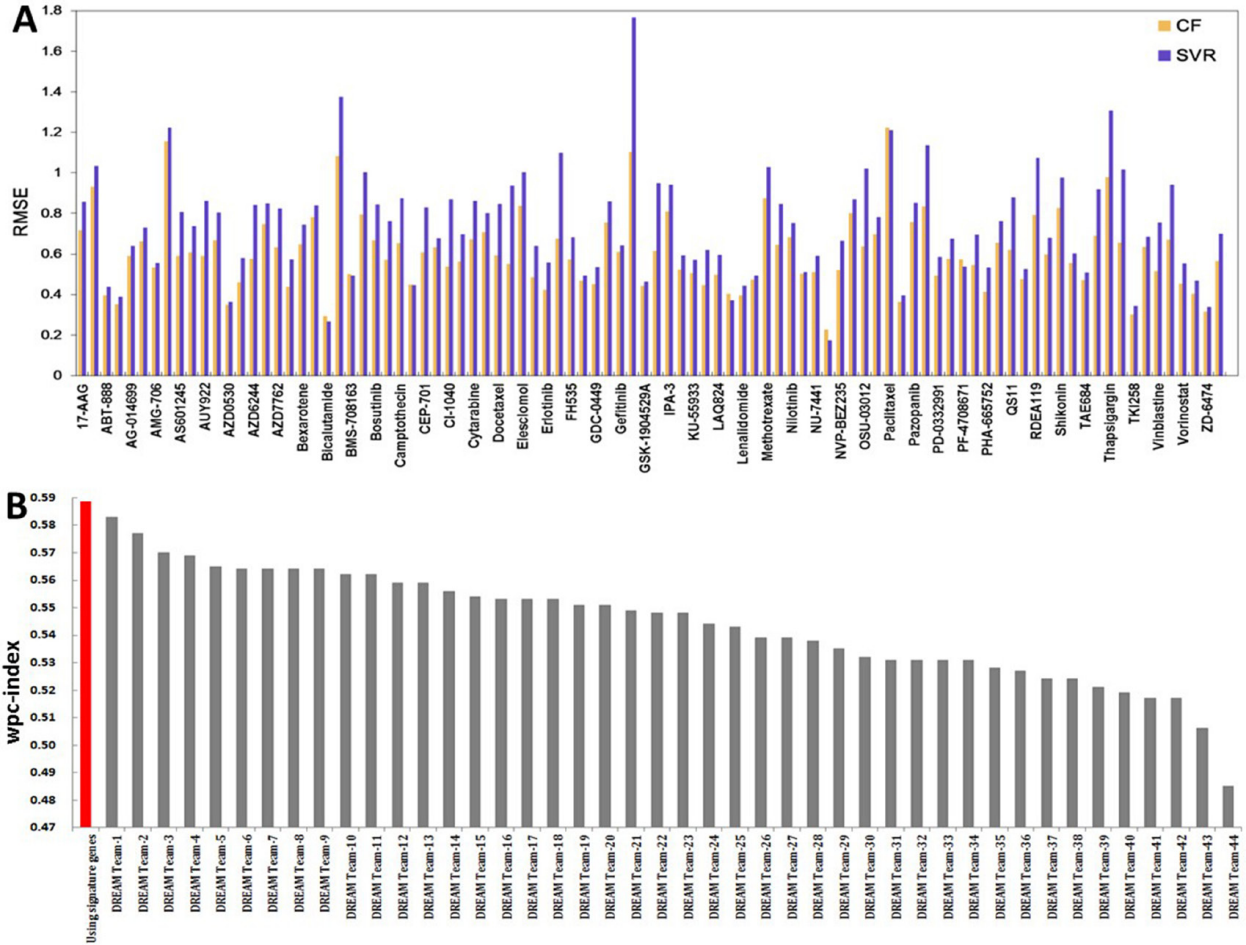
Performance assessment (**A**) Performance comparison between SVR and collaborative filtering based drug sensitivity predictions using gene expression profiles. (**B**) Performance of collaborative filtering based sensitivity predictions compared with the NCI-Dream challenge based on the wpc-index.

First, in order to evaluate the efficiency of our collaborative filtering based drug response prediction model, support vector regression (SVR) [26] was used as the baseline method for comparison (See additional file 1 for the description of SVR). We noted that the collaborative filtering based method consistently outperformed SVR on almost all tested drugs, and showed an average RMSE (Root mean squared error) that was 17% lower than SVR in the response prediction of 94 collected drugs by using transcriptional profiles (Figure 2A). RMSE is frequently used to measure the differences between values predicted by a model or an estimator and the values actually observed. Here, RMSE was used to assess the difference between the observed drug response measures (IC50) and the output of the fitted model (a lower RMSE value indicating better performance of the fitted model) [26].

Furthermore, Group Sparse Lasso was used to derive signature genes relevant to drug responses of a given cancer type based on transcriptional information (See Materials and Methods). By using signature genes, we tested our prediction method on data released from the NCI-DREAM drug sensitivity prediction challenge [6]. As all the cell lines involved in the NCI-DREAM challenge are breast cancer cell lines, the gene expression of the drug response signature genes derived from breast cancer cells were used to perform the prediction. Predictions from 44 different algorithms were experimentally assessed [6], and our method surpassed all 44 algorithms. As shown in (Figure 2B), our collaborative filtering model obtained a weighted probabilistic concordance-index (*wpc*-index) [6] of 0.589, while the best performed Bayesian multitask kernel learning (MKL) method from the DREAM challenge obtained a *wpc*-index of 0.583. The concordance index (c-index) is a nonparametric scoring method that provides a measure of similarity between two lists of measurements or ranks [6]. The above results confirmed that our model is very promising in predicting drug sensitivities in cancer cell lines.

### Signature genes related to drug sensitivity

**Figure 3:**
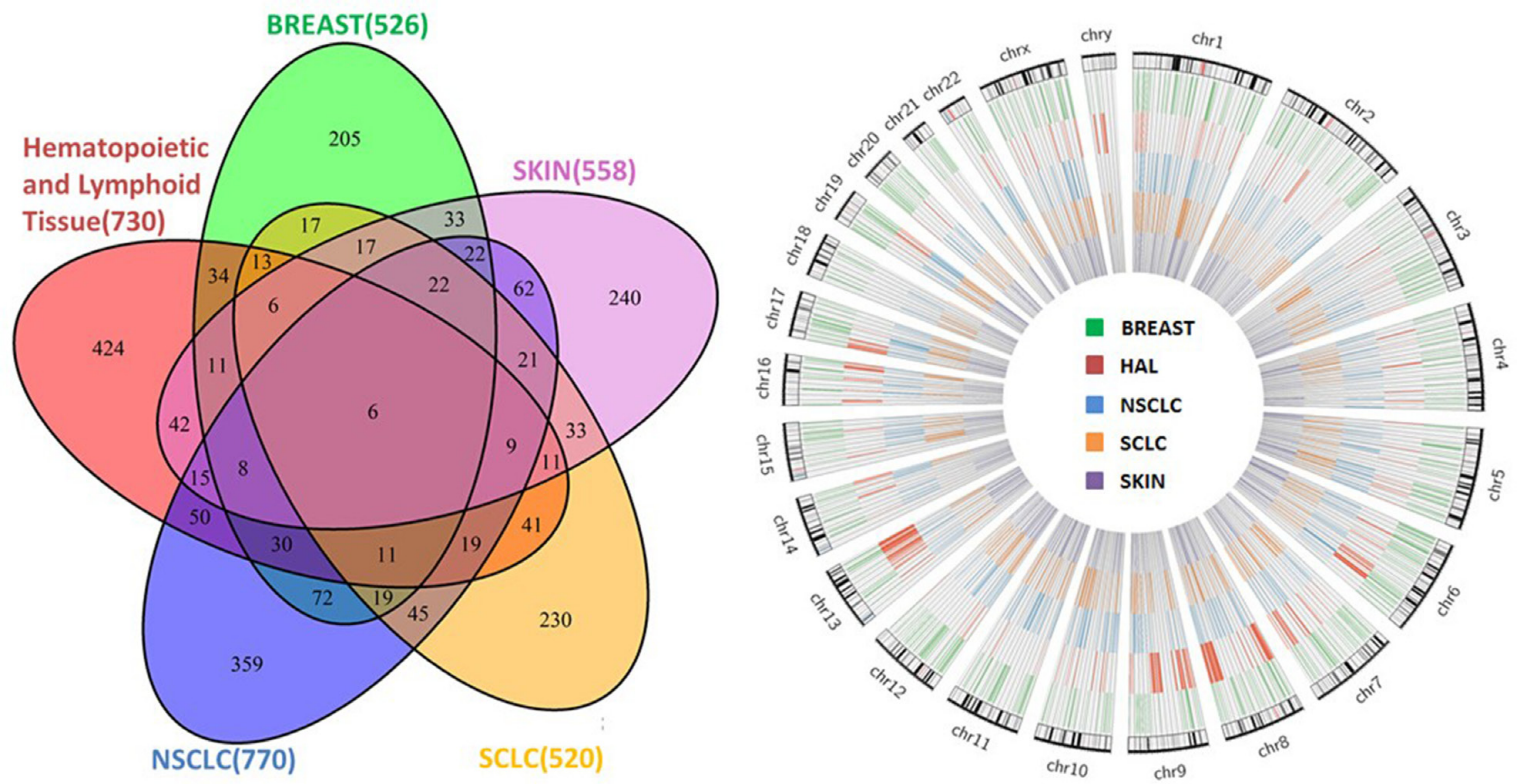
Overlap and genomic distribution of signature genes as related to drug sensitivity

Signature genes of drug sensitivity were derived for five different cancer types respectively using Group Sparse Lasso (Additional file 2): breast cancer (526 genes), hematopoietic and lymphoid cancer (730 genes), small cell lung cancer (SCLC; 520 genes), non small cell lung cancer (NSCLC; 770 genes), and skin cancer (558 genes). As shown in Figure 3, the signature genes selected for the five cancer types were identified to have no specific enrichment distribution in a particular chromosome. In addition, we also found that “*Ribosome*” was the most significantly enriched pathway for the signature genes of breast cancer, SCLC, NSCLC and skin cancer (Additional File 3). We also noticed that a large proportion of the signature genes were specific to only one of the five cancer types (Figure 3): 38.97% for breast cancer (205 genes), 58.08% for hematopoietic and lymphoid cancer (424 genes), 44.23% for SCLC (230 genes), 46.62% for NSCLC (359 genes), and 43.01% for skin cancer (240 genes). “*Complement and coagulation cascades*”, “*Hematopoietic cell lineage*”, “*Histidine metabolism*”, and “*ECM-receptor interaction*” are the pathways that were significantly enriched by signature genes specific to breast cancer, hematopoietic and lymphoid cancer, NSCLC and skin cancer respectively, while there were no significantly enriched pathways found for SCLC (Additional File 3). “*Complement and coagulation cascades*” play an important role in immune response. The complement system as a main column of innate immunity and the coagulation system as a main column in hemostasis undergo massive activation soon after injury. Complement activation could potentially be a very important event in anti-cancer immunity and immunotherapy as it may not only help with tumor clearance but also promote tumor growth [27, 28]. Coagulation disorders are common in neoplastic patients. A hypercoagulable state may be induced when malignant cells interact directly with a hemostatic system and activate the coagulation cascade. Thrombin is formed by the proteolytic cleavage of coagulation factor II in the coagulation cascade and acts, in turn, as a serine protease that converts soluble fibrinogens into insoluble strands of fibrin, and catalyzes many other coagulation-related reactions. It has already been reported that thrombin could support tumor cell malignancy [29]. Ana-Teresa et al. studied 12 candidate genes that are implicated in the etiology of breast cancer and found these genes were functionally involved in the complement and coagulation cascades pathway [30].

All the significant gene sets identified in this step provided an initial data set to analyze the cell line characteristics correlated with drug sensitivities for different cancer types. It should be noted that the current gene sets were identified using input cell line based drug response data. With the accumulation of more cell lines and drug test data, we believe that such gene sets will become more accurate to uncover drug response related mechanisms. Although the former comparison with NCI-DREAM validated, to some extent, the rationality of selected genes from our study, more comprehensive experimental validations are needed going forward. The goal of this study was not to validate our identified gene sets, but rather to provide an efficient approach for identifying the gene sets. Nevertheless, we also discussed these genes below based on various other analyses.

### Functional association of signature genes with synthetic lethality

**Figure 4:**
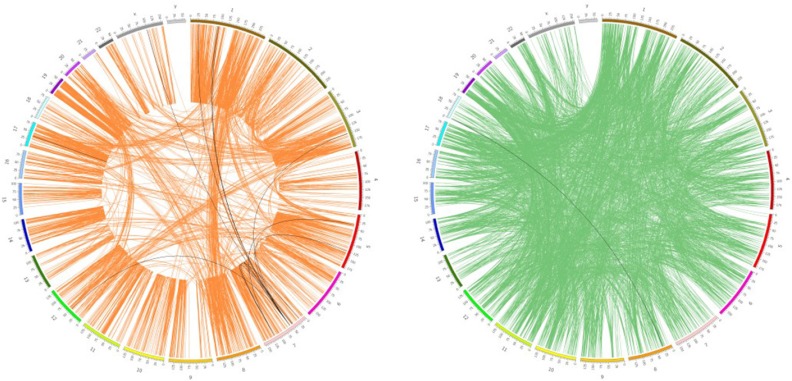
Genomic distribution of synthetic dosage lethality and synthetic lethality The circle with orange lines represents the SDL pairs, while the one with green lines represents the SL pairs, as documented in the work of Livnat Jerby-Arnon [26]. The black lines were SDL/SL pairs mapped to the targets of the 94 drugs and the related signature genes.

A large body of evidence points out that the onset of synthetic lethality (SL) may provide a useful tool for overcoming drug resistance to anticancer regimens. Here, we mapped the targets of the 94 collected drugs and the selected signature genes to a well curated dataset of the SL gene pairs and synthetic dosage lethality (SDL) gene pairs, as presented in the work of Livnat Jerby-Arnon [31] (See Materials and Methods). Only 21 non-redundant SDL pairs (from 6 drugs) and 1 non-redundant SL pair (from 1 drug) overlapped with the gene pairs, where gene A comes from the drug targets and gene B comes from the signature gene sets (See Additional file 4). Most of the paired genes were distributed on different chromosomes, while only a few of them were located on the same chromosome (Figure 4). In most of these SDL pairs gene B shows a higher expression value in the sensitive cells than in the resistant ones. The scarcity of mapped results such as these was probably due to several key facts:(1) Our initial drug sets are relatively small (94 drugs), and their targets (obtained from DrugBank) only account for a very small proportion of the SDL and SL genes; (2) The identification of SL and SDL pairs is still an open question, and currently there is no golden standard gene pair list in existence. Finally, (3) the synthetic lethality/synthetic dosage lethality is only one possible mechanism of drug sensitivity among various others, thus it is not surprising that we only mapped a few of the genes we identified to the background SDL and SL gene sets. Nevertheless, this analysis indicated that correlating drug targets and drug sensitivity related genes with SDL or SL pairs, may provide new clues to uncover drug sensitivity related mechanisms. One validated result in this study was a mapped gene pair among these SDL pairs, i.e., *EGFR* (Gene A) and *IGFBP3* (Gene B) correlated to Gefitinib sensitivity. It has been reported that *IGFBP3* is under-expressed in Gefitinib-resistant cells, and the addition of recombinant *IGFBP3* restored the ability of Gefitinib to downregulate PI3K/AKT signaling and inhibit cell growth [32]. This indicates that the overexpression of *IGFBP3* could induce sensitivity to EGFR-TKI Gefitinib.

### The expression patterns of drug sensitivity related genes differ across various tumor sample subtypes

**Figure 5:**
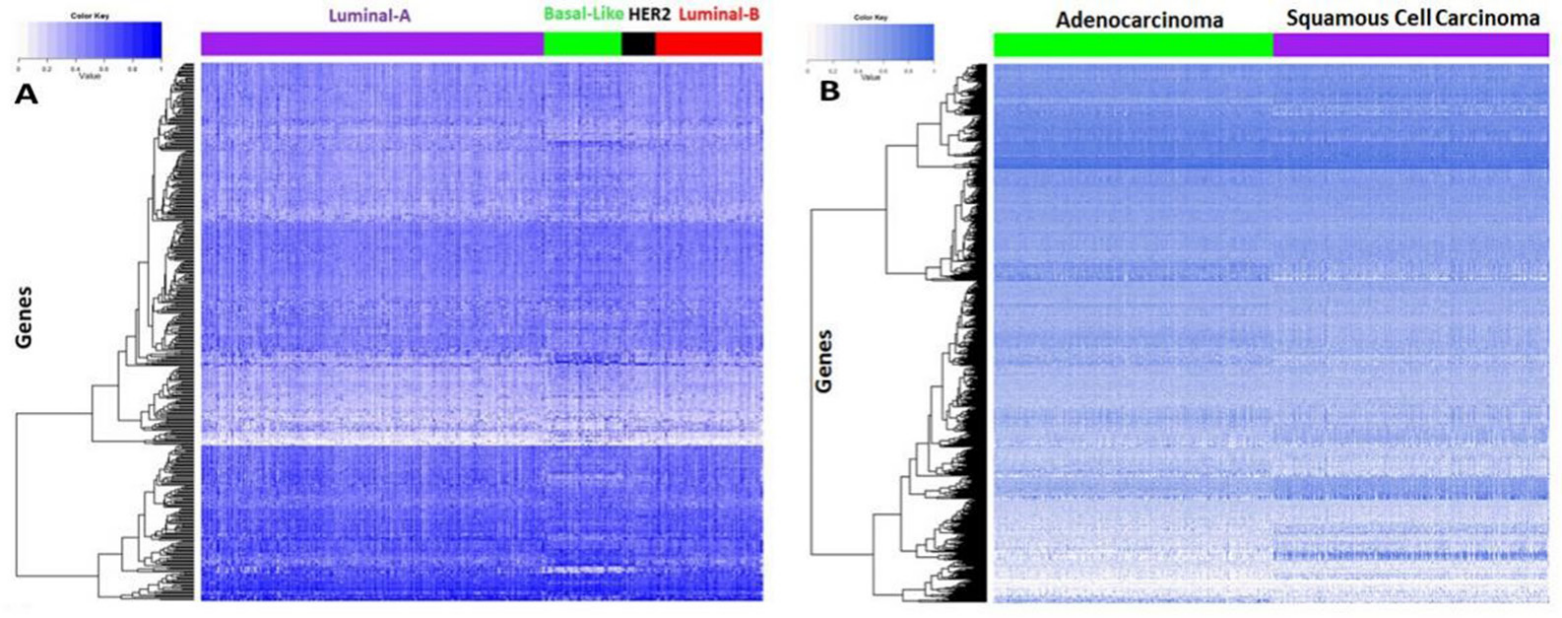
Tumor sample clustering using the identified drug sensitivity related genes (**A**) Tumor sample clustering of breast cancer. (**B**) Tumor sample clustering of NSCLC.

In this study we aimed to investigate the implications of the drug sensitivity related genes identified in the *in vitro* cell line systems for tumor samples. The gene expression data of tumor samples were retrieved from the TCGA for breast cancer and non-small cell lung cancer (NSCLC), given that other cancer types in our cell line study are not included in the TCGA. The signature genes selected for the two types of cancer cells were separately used to cluster the breast tumor samples and the NSCLC samples. We performed an unsupervised hierarchical cluster analysis for breast tumor samples using signature genes related to drug sensitivity in breast cancer cells. From Figure 5A, one sees that the signature genes showed different expression patterns across the four mRNA-expression subtypes: luminal A, luminal B, HER2E, and basal-like. Similarly, after conducting the unsupervised hierarchical clustering for NSCLC, the expression patterns of the signature genes for NSCLC also revealed obvious differences between adenocarcinoma and squamous cell carcinoma samples (Figure 5B). Generally, different drug treatments are effective for different subtypes of cancer, which implies that patients with different tumor subtypes respond differently to the same medication. This indicated that our selected signature genes derived from cancer cells could be used to classify the tumor patients, and could be extended to predict clinical responses to drug treatments *in vivo*.

### Signature genes related to drug primary resistance

Based on the selected signature genes related to drug sensitivities, we further screened the drug primary resistance related genes based on the integration of gene expression profiles with CNV. Our basic assumptions for primary drug resistance gene screening were that: (1) Drug resistance genes were also drug response related. In other words, we screened drug resistance genes from the former selected signature genes related to drug sensitivities (responses); (2) for each drug tested on multiple cell lines for a specific cancer type, we sorted the cell lines according to their response values (measured in pIC50) in an ascending pattern, and the expression levels of the drug resistance genes in the top activity as well as the bottom activity of the cell lines needed to be well-correlated with the corresponding cell line activities. This can be achieved by, first, making discretization of the cell line activity data to automatically categorize the cell line as sensitive, moderate or resistant to the drug perturbation. Then the spearman correlation of the expression levels of the genes in the sensitive and resistant cell lines with the activity of the sensitive and resistant cell lines can be calculated; (3) the CNV levels of the drug resistance genes in the top activity as well as the bottom activity of the cell lines needed to be well-correlated with the corresponding cell line activities.

Based on these criteria, for each drug we identified signature genes related to primary drug resistance as listed in Additional file 5. We further analyzed the functions of these genes in the following section.

### Downregulated *TOP2A* as a potential indicator for docetaxel drug resistance in breast cancer patients

**Figure 6:**
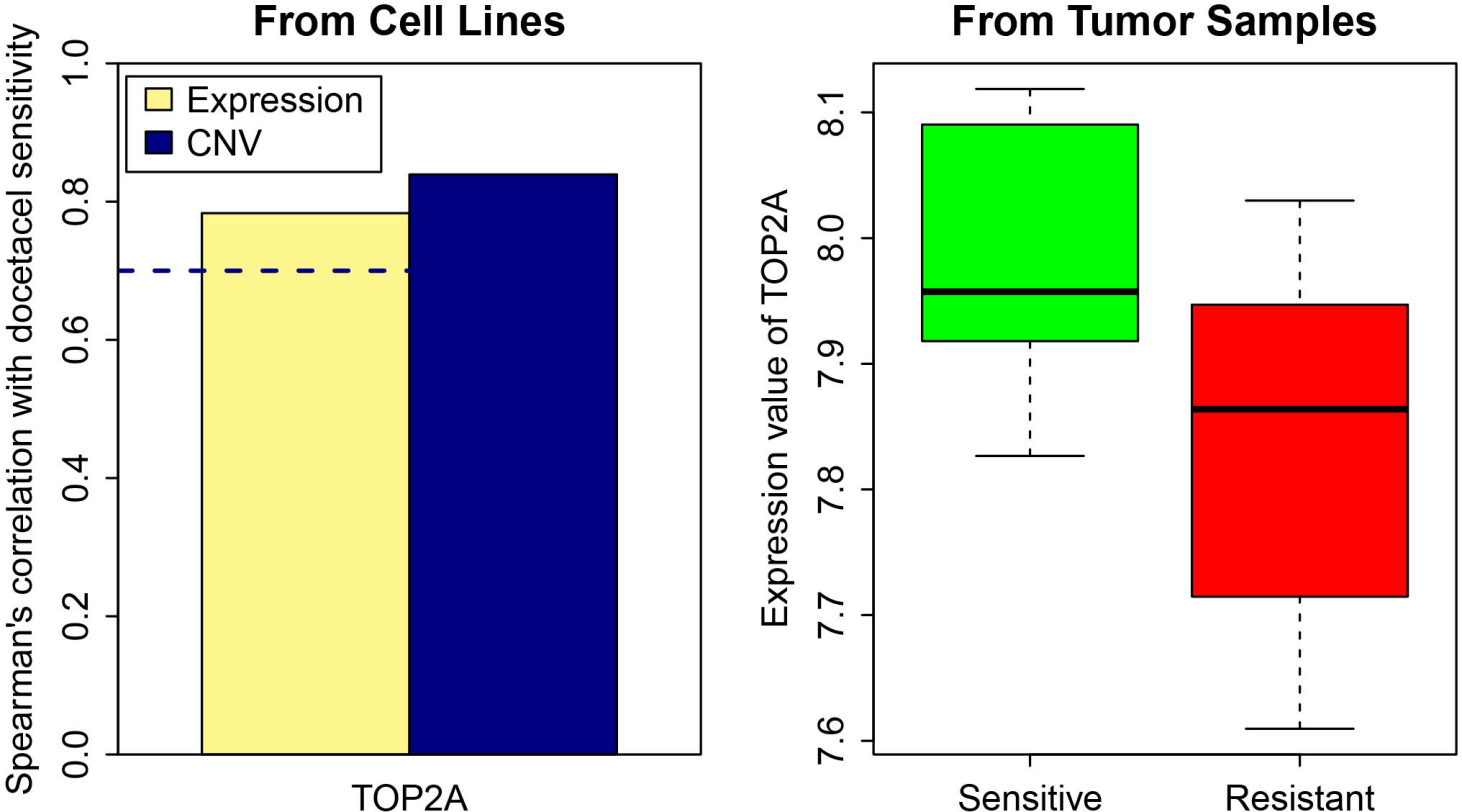
TOP2A gene expression and CNV in resistant and sensitive cell lines as well as in tumor samples The dash line represents spearman correlation cutoff: 0.7

To investigate the implications of the genes identified in *in vitro* cell line systems for patient samples, we collected gene expression microarray data obtained from 24 breast cancer tumor biopsies through a clinical trial, which measured the responses of patients to docetaxel neoadjuvant treatment [33]. Based on our previously identified drug resistance related signature genes for breast cancer cell lines, we identified *TOP2A* as a potential indicator of drug response to docetaxel in breast cancer. In our cell line analysis, the expression and CNV of *TOP2A* demonstrated a high correlation with the cell line activity profile for the drug docetaxel. *TOP2A* was downregulated in resistant tumor samples in contrast to sensitive samples (Figure 6). A previous study on NSCLC A549 cells reported that the induction of apoptosis by docetaxel requires DNA replication, and replication-medicated double-strand breaks (DSBs) are critical triggers of docetaxel-induced apoptosis [34]. *TOP2A* encodes the key enzyme in DNA replication and makes DSBs. This further supports the potential role of *TOP2A* in drug response to docetaxel in breast cancer cells. Interestingly, *TOP2A* presented the same trend of gene expression change between the sensitive groups and resistant groups of the tumor patients as observed in the docetaxel sensitive and resistant cancer cells. This indicates that *TOP2A* may be a marker gene relevant to docetaxel resistance in breast cancer patients.

### Genes associated with cell communication are potentially relevant to cisplatin resistance in breast cancer

**Figure 7:**
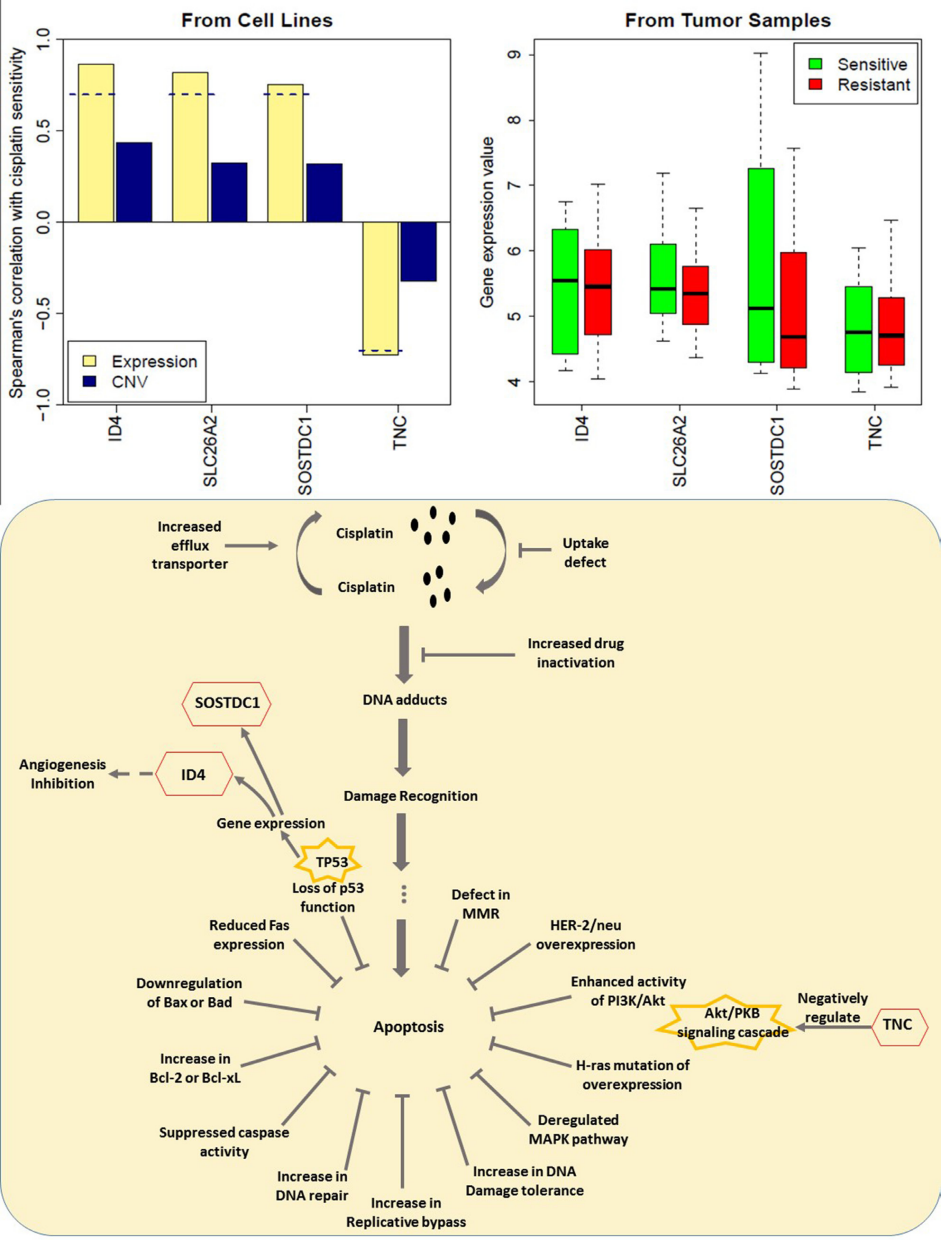
Gene expression of 4 genes in the resistant and sensitive cell lines as well as in TNBC tumor samples The implications for the potential role of *ID4*, *SOSTDC1* and *TNC* in cisplatin – resistance were presented. The expression value was log2-transformed. The dash line represents spearman correlation cutoff: 0.7

A similar process was performed on the gene expression data from 28 women with stage II or III triple-negative breast cancer (TNBC) [35]. The Miller-Payne scoring system was used to assess tumor responses after four cycles of cisplatin at 75 mg/m^2^ every 21 days. Four marker genes (*ID4, SOSTDC1, SLC26A2, TNC*) relevant to drug activity to cisplatin on breast cancer cells were derived from the signature genes for breast cancers (Figure 7). Upregulated *ID4, SOSTDC1, SLC26A2* and downregulated *TNC* may play a role in regulating drug resistance. Among the genes presented, we found that *TNC* were involved in cell adhesion and cell communication which has been linked to cisplatin-induced cell death [36]. The mechanisms responsible for cisplatin resistance were only reported sporadically, which included the loss of p53 function, overexpression of HER2/neu, activation of the PI3K/AKT pathway etc [37]. The indicator role of *SOSTDC1, ID4* and *TNC* for cisplatin resistance may lie in the loss of p53 function and the activation of the PI3K/Akt pathway. We were the first group to summarize the potential role of *SOSTDC1, ID4* and *TNC* in cisplatin-resistance, as shown in (Figure 7B). It can be seen that the gene expression of *SOSTDC1* and *ID4* is regulated via the TP53 pathway in breast cancer [38–41], and that *ID4* may influence the expression of proangiogenic cytokines, such as *IL8* and *GRO-alpha*, increasing the angiogenic potential of cancer cells [39]. [43, 44] Besides, *TNC* was found to be a negative regulator of the AKT/PKB signal transduction pathway [42]. However, the role of *SLC26A2* in cisplatin resistance is still unclear. Given that these genes showed accordant trends of change in gene expression values in the TNBC patients as in the breast cancer cells (Figure 7), they may also be relevant to cisplatin resistance in TNBC patients.

### Genes modulating PI3K pathways demonstrate a consistent trend of expression change in both primary and acquired doxorubicin resistance

**Figure 8:**
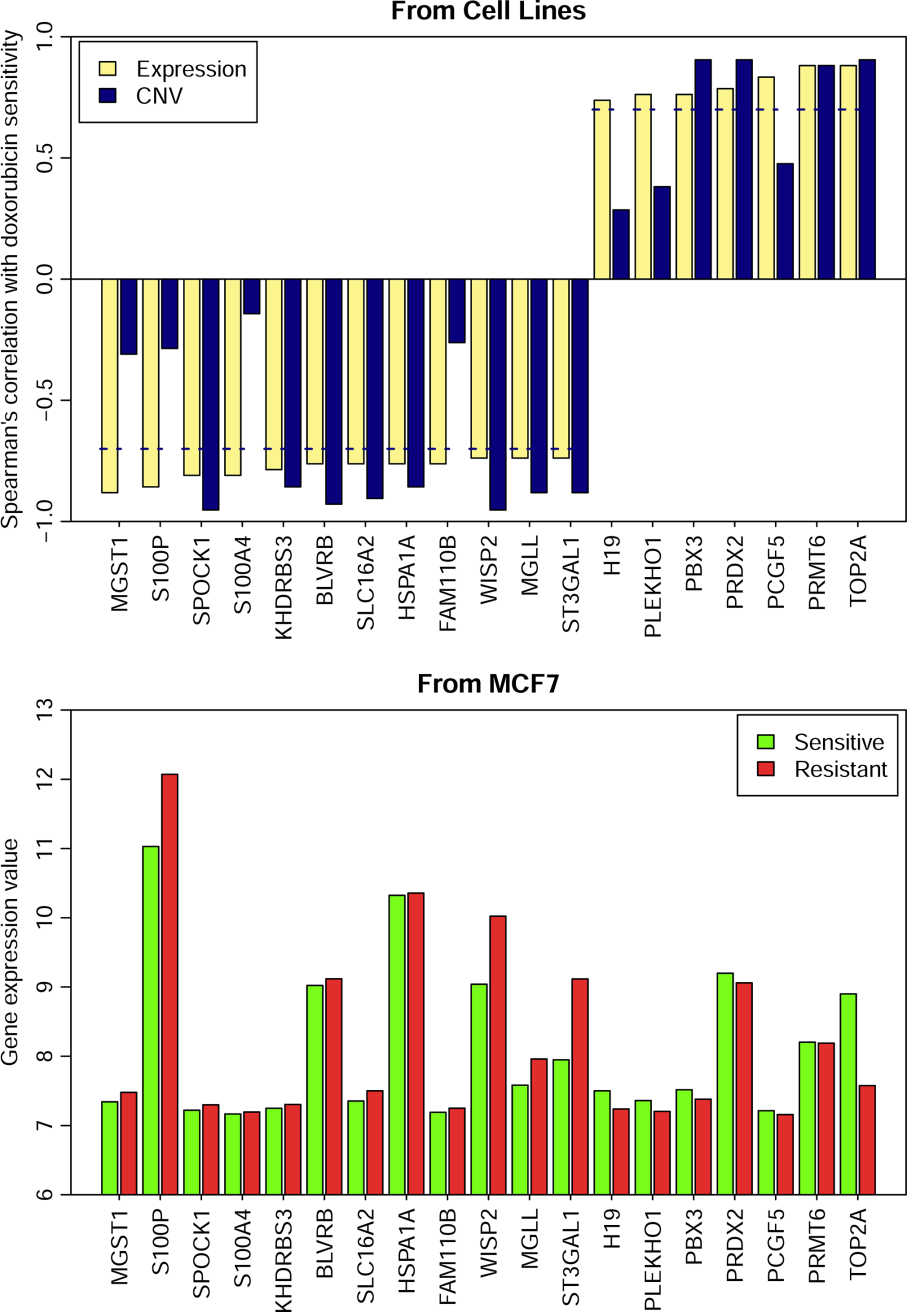
Gene expression and the CNVs of 19 genes in the resistant and sensitive cell lines The dash line represents spearman correlation cutoff: 0.7

Doxorubicin is a naturally occurring anthracycline antibiotic that is broadly considered the most active single agent available for the treatment of breast cancer [43]. On the other hand, cancer drug resistance limits its use, and *ABCB1(MDR1, P-gp), ABCC1 (MRP1)* as well as other transporters have been characterized in previous studies for their roles in drug resistance [44]. In our study, 19 genes were identified as potential markers associated with primary resistance to doxorubicin in breast cancer cells (Figure 8). Among the 19 genes, *TOP2A* showed a higher expression value and higher CNV values in breast cancer cells sensitive to doxorubicin than those in the resistant cells, while *S100P* and *S100A4* showed lower expression values and lower CNV values in the sensitive cells than those in the resistant ones. Doxorubicin induces cancer cell death by many mechanisms, the most notable is *Top2A* poisoning, and also the down-regulation of *Top2A* is obviously detected in doxorubicin resistant MCF7 cells [45]. Overexpression of *S100P* and *S100A4* has been described in doxorubicin-resistant cell lines [46, 47]. *BLVRB*, *MGST1* and *H19* have been linked to multidrug resistance, including resistance to doxorubicin [48–50]. *H19* has been found to increase cellular doxorubicin accumulation levels via suppressing *MDR1/P-glycoprotein* expression which is important in decreasing doxorubicin accumulation levels [50]. Additionally, *ST3GAL1* encodes asialytransferase which participates in the sialylation that is associated with doxorubicin resistance [51]. A possible mechanism responsible for sialytransferase -related doxorubicin resistance may be due to the PI3K/AKT signaling pathway. The manipulation of the sialytransferase genes’ expression modulated the activity of the PI3K/AKT signaling pathway and its downstream target, thus regulating the proportionally mutative expression of P-glycoprotein (P-gp) and MDR-related protein 1 (*MRP1*) [51]. *PRMT6* may also relate to the PI3K/AKT mechanism via *PTEN*. *PTEN* is a tumor suppressor gene that inhibits the PI3K pathway, and lower *PRMT6* expression may result in increased *PTEN* expression, decreased cell cycle progression and increased breast cancer cell apoptosis [52].

## DISCUSSION

In the current study, we collected a comprehensive compilation of drug-cell line response data from a large panel of cancer cell lines. Leveraging this dataset, we achieved the following three goals: (1) we designed a novel and efficient *in-silico* pipeline to identify signature genes that may be relevant to drug sensitivity, and that further identifies drug resistance genes for different cancer types, by using an integrative analysis of gene expression profiles with copy number variations (CNVs) of cell lines as well as their *in vitro* drug responses; (2) we presented a novel collaborative filtering based drug sensitivity prediction model, which outperformed the 44 algorithms submitted to the DREAM competition using breast cancer cell data; (3) the functions of the identified signature genes related to drug sensitivities were carefully analyzed at the pathway level, the synthetic lethality level, as well as applied to the classification of the different subtypes of the TCGA tumor samples. Additionally, our *in-silico* pipeline has shown promise in: (1) translating genomic data into customized maker genes relevant to the resistance of specific drugs in individual patients with a specific cancer type; and (2) serving as the bridge to explore common mechanisms between primary resistance and acquired resistance to a given drug.

Testing a large number of cell lines and compounds is one important prerequisite for achieving remarkable power in predicting drug responses. Here, we curated a large compilation of drug-cell line response information, consisting of 94 drugs on 608 cancer cell lines. Using the simultaneous modeling of drug responses across all the drugs and cell lines from the large dataset, our *in-silico* pipeline, which incorporates collaborative filtering based algorithms together with Group Sparse Lasso, achieved superior performance in predicting drug responses and identifying signature genes that may correlate with drug sensitivity and drug resistance. This model allows for the integration of different data sources unlike traditional statistical models. According to the DREAM competition, gene expression microarrays consistently provided the best predictive power among the individual profiling data sets of DNA copy-number variations, transcript expressions, mutations, DNA methylation and protein abundance [6]. When applying our *in-silico* pipeline to the selected signature genes with relevant drug sensitivities for breast cancer, we achieved a *wpc-index* of 0.589 in the DREAM competition, which was superior to all the other reported approaches.

A great disparity was observed in the signature genes identified for the 5 different cancer types, which indicates that genes relevant to drug resistance are highly dependent on the disease type. As somatic mutations and genomic plasticity associated with cancer are the foundation of drug resistance [22], cancer heterogeneity explains why every cancer expresses a different array of genes relevant to drug sensitivity and resistance. Furthermore, we also found that the selected signature genes for breast cancer and NSCLC show distinct expression patterns across the different subtypes of the TCGA tumor samples.

Based on previous studies which have demonstrated that DNA CNVs are influential factors in altered gene expression levels in cancer, CNV information on the corresponding cancer cells was integrated with the selected signature genes to help identify these potential maker genes that may correlate to resistance of a specific drug in a specific cancer cell type. To our knowledge, this is the first time that baseline copy number variation has been integrated with the gene expression of cancer cell lines in identifying maker genes relevant to drug resistance. The marker genes identified for each drug were quite different, and this also implies that genes relevant to drug resistance are highly drug-specific. The current limitation of our method is that we can only identify genes relevant to drug resistance in the pre-collected drugs, totaling 94 at present. Nevertheless, with the rapid accumulation of high-throughput experiments such as the LINCS project, our method will eventually be applied to massive compound data.

The potential indicator role of the genes in resistance to the corresponding drugs was also explored in the clinical cancer patients. For docetaxel, a well-established clinically anti-mitotic chemotherapy medication that works by interfering with cell division, the gene *TOP2A*, which encodes the key enzyme involved in DNA replication was proposed as the marker gene most relevant to docetaxel-resistance in breast cancer patients. In addition, the potential maker genes relevant to cisplatin-resistance in TNBC patients were involved in cell communication, which has been correlated with cell death induced by cisplatin [36]. Although the effectiveness of these indicator genes needs further validation, our *in-silico* pipeline provides the opportunity to translate genomic data derived from baseline cancer cell lines into actionable, and individualized maker genes relevant to the resistance of specific drugs in clinical applications.

In addition to primary or intrinsic resistance, acquired resistance also exists. Primary resistance is a pre-existing resistance that is present prior to the exposure to a given drug. In contrast, acquired resistance develops in tumors that were initially sensitive to the drug, after exposure to this drug [53]. In this work, a comprehensive compilation of baseline data of cancer genomes and drug-cell line response data were explored with the ultimate aim to characterize the mechanism of primary resistance. Unfortunately, the long-term effectiveness of these drugs is hindered by the development of drug resistance due to the mutation of the targeted protein, the amplification of alternative oncogenes, or the inactivation of alternative survival pathways [54]. Taking doxorubicin as an example, we attempted to explore the association between primary resistance and acquired resistance to drugs. Genes modulating PI3K pathway showed a consistent trend of gene expression changes, be it in a primary resistance or acquired resistance setting for doxorubicin. These genes may be the gateway to explore the association between primary resistance and acquired resistance to doxorubicin.

Finally, the rational of our identified drug sensitivity or resistance related genes remains to be further validated by more comprehensive experimental tests, while the basic goal of the current study was to provide an efficient framework for identifying such genes based on the accumulated *in vitro* cell line and drug perturbation data, rather than the validation of the identified genes. Such methods will prove invaluable in the exploration of cancer resistance mechanisms when, in the near future, we will be able to access millions of drug perturbation data generated from *in vitro* cell line systems and the individual patients receiving personalized medical care.

## MATERIALS AND METHODS

### Data collection

The gene expression, copy-number variation (CNV), and cancer cell line mutation data was retrieved from a website compiling genomic information on 947 human cancer cell lines, namely the Cancer Cell Line Encyclopedia (CCLE) [3]. We used gene expression data from Affymetrix U133 Plus 2.0 Arrays, CNV data from Affymetrix SNP6.0 arrays, and Oncomap mutation data. To exclude inconsistencies among different data sets and/or different platforms, gene expressions, CNVs, the mutation data of cancer cell lines from other studies were not used in this work.

Drug information and drug response measures (IC50) were retrieved and curated from the CCLE website together with two other large pharmacogenomic studies, including the Cancer Genome Project (CGP): Genomics of Drug Sensitivity in Cancer (GDSC) [5], and the NCI-DREAM project [6]. IC50 measurements across all studies (IC50 represents the concentration of a drug that is required for 50% inhibition *in vitro*) were all set to pIC50 = −log_10_ (IC50M). In total, there were 168 drugs tested on 608 cancer cell lines with all three genomic data types of cancer cell lines available.

It should be noted that the IC50 data of some of the 168 drugs were scattered across multiple cancer types. Drugs missing too much IC50 data (with missing data on > 40% of the 608 cancer cell lines) were removed. Finally, 94 drugs were included for further study. All of these data are available in Additional file 6.

### A general pipeline for drug sensitivity prediction and signature gene identification

Once the baseline cancer genome and drug response data were carefully collected and curated, an efficient *in-silico* pipeline was set up to identify the signature genes relevant to drug sensitivity and resistance for different cancer types. A collaborative filtering based algorithm [25] was applied to predict drug sensitivities in cancer cell lines, and Group Sparse Lasso was applied to select the signature genes of drug sensitivities for a specified cancer type. The particular reasons for designing such a pipeline included their highly accurate prediction rates, simultaneous integration of multiple drug-cell line data and the scalability for further significant gene identification [53]. We further conducted a comprehensive analysis of the identified signature genes using pathway enrichment analysis, synthetic lethality analysis, and validated these signature genes using TCGA clinical patient data. We screened the signature genes that may correlate with the primary resistance of a specific drug on a specific cancer cell type by incorporating CNV information with the outlier detection, cell line activity discretization and spearman correlation analysis.

### Predicting drug sensitivity based on a collaborative filtering method

Collaborative filtering [25] was incorporated in this work to predict drug sensitivities in cancer cell lines and compare them with the methods from the NCI-DREAM challenge. The collaborative filtering algorithm has the advantage of simultaneously modeling heterogeneous drug-cell line sensitivities across multiple drugs. To implement our method, the following data matrices were provided: (i) a drug sensitivity matrix with −log (IC50nM) information of 94 drugs on 608 cell lines, denoted as *S* S ∈ ℝ^94×608^; (ii) a similarity matrix of cell lines measured as the cosine similarity of features of cell lines (gene expression profiles of cell lines), denoted as $W^{C}=\left[W_{ij}^{c}\right]\in {\mathbb{R}}^{608\times 608}$; (iii) a similarity matrix of drugs measured using Tanimoto coefficient [55, 56] based on drug features (881 binary fingerprint from PubChem), denoted as $W^{D}=\left[W_{ij}^{D}\right]\in {\mathbb{R}}^{94\times 94}$. Then, a matrix factorization based model was applied to solve two matrices $U\in {\mathbb{R}}_{+}^{94\times k}$ and $V\in {\mathbb{R}}_{+}^{608\times k}$ to represent *S* ≈ *UV^T^*, where *k* is the dimensionality of the low-dimensional representation. (See Supplementary Materials)

We tested our collaborative filtering model on the data released from the NCI-DREAM drug sensitivity prediction challenge [6]. Participants in the challenge were supplied with various profiling data for 53 breast cancer cell lines, and the drug response data of 35 cell lines for 28 compounds (training data). Participants were challenged to predict a ranked list of the most sensitive (to be ranked first) to most the resistant (to be ranked last) cell lines for each individual drug across all remaining 18 cell lines (testing data). The assessment of predictions was based on participant's ranking of all 28 therapeutic compounds across all 18 test cell lines. The same training data and testing data used in the DREAM competition were set for our collaborative filtering model. 7 cell lines were excluded due to missing gene expression profiles. Furthermore, the drug combination (4 − HC + Dox) and the antibody (Trastuzumab) were removed due to the unavailability of the 881 bit binary fingerprint from Pubchem. −log_10_ (GI50) was used as the dose-response value as it was used in DREAM algorithms. A weighted, probabilistic concordance-index (*wpc*-index) was used to evaluate the final performance of our collaborative filtering model.

### Identifying drug sensitivity related genes for a specified cancer type

Group Sparse Lasso was applied to select signature genes related to drug responses for specified cancer types, which was presented as a very robust identification feature in the machine learning community [57]. This method can be seamlessly incorporated into the collaborative filtering based cell line response prediction model, and can be used for feature selection across multiple drug samples. In the aforementioned part of response prediction, we have obtained two matrices $U\in {\mathbb{R}}_{+}^{94\times k}$ and $V\in {\mathbb{R}}_{+}^{608\times k}$ to represent the sensitivity matrix as *S* ≈ *UV^T^*. In the Group Sparse Lasso process, with corresponding feature matrices *F^D^* ∈ ℝ ^94×*h*_1_^ and *F^D^* ∈ ℝ ^*l*×*h*_2_^, where *h*_1_ is the length of Pubchem fingerprint and *h*_2_ represents the amount of genes in the expression profile, *l* is the number of cell lines for a specific cancer type. The feature selection task for the drug structure or genes on the whole genome could be treated as the *l*_1_ / *l_q_* -norm regularized multi-class least squares problem, which resulted in two sparse feature weight matrices *P* ∈ ℝ*^h_1_×k^* and *Q* ∈ ℝ *^h_2_×k^*, where *U* ≈ *F^D^ P* and *V* ≈ *F^C^Q*. There were several rows in these two feature matrices with zero elements, indicating that the corresponding features were not important and were not selected (See Supplementary Materials).

### Pathway analysis

Gene set enrichment analyses were performed for the functional annotation of the gene signatures for the 5 cancer types. Functional Annotation Tools in DAVID Bioinformatics Resources [58] were used to carry out these analyses. KEGG pathways with *p*-values of less than 0.05 and more than two genes were considered significantly enriched functional pathways and used for further analysis.

### Synthetic lethality analysis

Synthetic lethality (SL) occurs when the inhibition of two genes is lethal while the inhibition of each individual gene is not. Gene A and gene B form an SL pair if the inactivation of gene A renders the essentiality of gene B, while the two genes form a synthetic dosage lethality (SDL) pair if the over-activity of gene A renders gene B essential. In the work of Livnat Jerby-Arnon [31], SL and SDL interactions in cancer were identified by analyzing large volumes of cancer genomic data. We mapped the targets of the 94 collected drugs to gene A, and fished out the corresponding gene B from the identified SL and SDL interactions by Livnat Jerby-Arnon et al. Then, those SL and SDL pairs remained in drug targets (gene A), and signature genes (gene B).

### Clustering of the tumor samples using drug sensitivity related genes from cancer cells

Gene expression data of breast cancer and non-small cell lung cancer (NSCLC) tumor samples were retrieved from TCGA. In total, gene expression profiles were curated for 396 breast tumor samples with prior knowledge of their mRNA-expression subtypes: luminal A, luminal B, HER2E, and basal-like. Gene expression profiles were also collected for 540 samples and 548 samples of squamous NSCLC patients and adenocarcinoma NSCLC patients respectively. First, the gene expression data on breast tumor samples was picked out for the signature genes derived from breast cancer cell lines. Then, the gene expression data was used to perform unsupervised hierarchical clustering of these tumor samples. The NSCLC samples were clustered in a similar way. It should be noted that the gene expression data on tumor samples was not available for all of the selected signature genes (Additional file 7). As this only accounts for a small proportion of the signature genes, the gene expression pattern for the tumor subtypes would barely be affected.

### Identifying potential drug resistance genes for a specific drug in a specific cancer type

#### Outlier detection

As the main purpose of this study was to investigate drug resistance with the cell line expression profiles, interference from CNVs and mutations was excluded. An outlier detection process was designed to make sure that the cancer cell lines kept a concordant CNV and mutation background but with different expression backgrounds. Outlier detection was performed on CNV and mutation data respectively. For each profile, a cell line was defined as an outlier if the Euclidean Distance to the geometrical center of these cell lines fell into the furthest 10% ranked ones and was removed after further analysis. It should be noted that all silent mutations were excluded, nor were they used to perform the outlier detection.

#### Automatic discretization of cell line activity data

For each drug tested on multiple cell lines for a specific cancer type, we sorted the cell lines according to their response values (measured in pIC50) in an ascending pattern, and the expression levels of the drug resistance genes in the top activity as well as the bottom activity of the cell lines needed to correlate well with the corresponding cell line activities. This can be achieved by firstly making discretization of the cell line activity data to automatically categorize the cell line into sensitive, moderate or resistant to drug perturbation. Such an automatic discretization was performed by an *Qualitative Representation* method, which was first introduced by us to address the discretization of microarray data [59]. The basic idea of this *Qualitative Representation* is to represent the cell line activity in a qualitative or semi-quantitative manner by considering the whole distribution of the activities (Detailed algorithm can be referred in [59]. As a result, the qualitative representation of the cell line activity is composed of signed integers and 0's, i.e. sensitive (+1), resistance (−1), or moderate (0). And all the subsequent analyses are conducted only for sensitive and resistance cell lines.

#### Screening resistance related genes by spearman correlation calculation

Drug resistance related genes were screened when the following criteria were satisfied:

1. The spearman correlation calculated between drug activity in the sensitive and resistant cell lines and the gene expression in the sensitive and resistant cell lines was > 0.7.
2. The genes should show the same tendency between the spearman correlation value of drug activity and the gene expression and that of drug activity and CNV (both positive or both negative).

### Clinical trial datasets

We collected clinical trial datasets that assessed tumor gene expression before drug treatment (using expression microarrays) and subsequently measured a clear drug response phenotype. Patients needed to have been treated with monotherapeutic drugs among the 94 collected drugs tracked in this study. Additionally, sensitivities to the particular drug needed to have been quantified on the cell lines of the same cancer type. Finally, we obtained two datasets with one for docetaxel [33] (GEO accession number: GSE349; GSE350) and one for cisplatin [35] (GEO accession number: GSE18864). Using these data, we attempted to check whether our signature genes derived from cancer cells could be used as marker genes associated with clinical drug responses.

### Datasets of acquired resistance

We also collected datasets of acquired resistance with gene expression profiles at different stages during the development of acquired resistance. As the tested drug needed to fall within the 94 collected drugs, we obtained only one eligible dataset for dororubicin [8] (ArrayExpress accession number: E-MTAB-1643). Human breast cancer MCF7 cells were selected for dororubicin resistance by first treating the cells with 1 μM dororubicin for 48 hours. The cells were then exposed to 100 nM dororubicin for 2 weeks. After this period, the surviving cells were resistant to further dororubicin treatment.

## SUPPLEMENTARY MATERIALS AND ADDITIONAL FILES










