# Supplementary material for: Identifying anti-cancer drug response related genes using an integrative analysis of transcriptomic and genomic variations with cell line-based drug perturbations
Source: Oncotarget. 2016 Jan 25;7(8):9404–19. doi: 10.18632/oncotarget.7012 (PMC4891048; doi:10.18632/oncotarget.7012)
Supplement: Supplementary file 1 [file oncotarget-07-9404-s001.pdf]

Identifying anti-cancer drug response related genes using an integrative analysis of transcriptomic and genomic variations with cell line-based drug perturbations

Supplementary Materials

Additional File 1: is a file introducing 2 methods: Collaborative filtering (CF) based drug sensitivity prediction and sparse group lasso for drug sensitivity gene selection.

Support vector regression (SVR) based drug sensitivity prediction

As a baseline drug sensitivity prediction model, support vector regression (SVR) is the version of SVM for regression, which was proposed in 1996 by Vladimir N. Vapnik, Harris Drucker, Christopher J. C. Burges, Linda Kaufman and Alexander J. Smola [1]. The model produced by support vector classification as commonly used in support vector machine (SVM) depends only on a subset of the training data, because the cost function for building the model does not care about training points that lie beyond the margin. Analogously, the model produced by SVR depends only on a subset of the training data, because the cost function for building the model ignores any training data close to the model prediction. Training the original SVR means solving:

$$\text{minimize } \frac{1}{2}\|W\|^2$$

$$\text{subject to } \begin{cases} y_i - < w, x_i > - b \leq \in \\ < w, x_i > + b - y_i \leq \in \end{cases}$$

where x_i is a training sample with target value y_i, in our study the cell line feature representation and the cell line sensitivity. The inner product plus intercept $\langle w, x_i \rangle + b$ is the prediction for that sample, and $\in$ is a free parameter that serves as a threshold: all predictions have to be within an $\in$ range of the true predictions. Slack variables are usually added into the above to allow for errors and to allow approximation in the case the above problem is infeasible.

Collaborative filtering (CF) based drug sensitivity prediction

In this study, we proposed a matrix factorization based collaborative filtering model [2] to predict the sensitivies of antic-cancer drugs on cancer cell lines, borrowing from the recommendation system concept which is prevalent in the computer science community [3]. Supposing that we have M anti-cancer drugs and N cancer cell lines, collaborative filtering represents the drug–cell line response relationship through the input of known drug-cell line response matrix $S \in \mathbb{R}^{M \times N}$, drug-drug similarity matrix $W^D = \left[W_{ij}^D\right]$ and cell line-cell line similarity matrix $W^C = \left[W_{ij}^C\right]$. $Y \in \mathbb{R}^{M \times N}$ is an indicator matrix to denote training and testing data. Then, the matrix factorization based model aims to seek two matrices $U \in \mathbb{R}_+^{M \times K}$ and $V \in \mathbb{R}_+^{N \times K}$ to represent $S \approx UV^T$, where k is the dimensionality of the low-dimensional representations. The method of graph regularized weighted nonnegative matrix factorization (GWNMF) minimized the following objective:

$$\begin{aligned} J_{GWNMF} &= \sum_{i=1}^{M}\sum_{j=1}^{N} Y_{ij}(S_{ij} - (UV^T)_{ij})^2 \\ &+ \frac{1}{2}\sum_{i=1}^{M}\sum_{j=1}^{N} \| d_{i.} - d_{.j} \|^2 W_{ij}^D \\ &+ \frac{1}{2}\sum_{i=1}^{M}\sum_{j=1}^{N} \| c_{i.} - c_{.j} \|^2 W_{ij}^C \end{aligned}$$

$$\begin{aligned} &= \| Y \odot \left(S - UV^T\right) \|_F^2 \\ &+ \lambda_1 \operatorname{tr}\left(U^T L_U U\right) + \mu \operatorname{tr}\left(V^T L_V V\right) \\ &\text{s.t } U \geq 0, V \geq 0 \end{aligned}$$

where $\odot$ is Hadamard product, $\|\cdot\|_F$ is Frobenius norm, u_i, $1 \leq i \leq N$ and v_j, $1 \leq j \leq M$ are the low-dimensional representations of anti-cancer drugs and cancer cell lines respectively, $\lambda_1, \mu \geq 0$ are regularization parameters. Similarity matrices W^D and W^C were

calculated as following, $F^D \in \mathbb{R}^{M\times h_1}$ and $F^C \in \mathbb{R}^{N\times h_2}$ are drug feature matrix and cell line feature matrix respectively, where h_1 is the length of Pubchem fingerprint and h_2 is the amount of genes in expression profile. The Tanimoto coefficient was utilized to calculate drug-drug similarity because anti-cancer drugs were described as 881 bit binary fingerprint. For cancer cell lines, Cosine distance was chosen as a similarity measurement in addition to two others– Person Correlation Coefficient and the reciprocal of Euclidean Distance, after a comprehensive comparison of their performances on a small scale expression data set.

Sparse group lasso for drug sensitivity gene selection

In the 1st part of sensitivity prediction, we have obtained two matrices $U \in \mathbb{R}_+^{M\times K}$ and $V \in \mathbb{R}_+^{N\times K}$ to represent sensitivity matrix as $S \approx UV^T$. With corresponding feature matrices $F^D \in \mathbb{R}^{M\times h_1}$ and $F^C \in \mathbb{R}^{N\times h_2}$, where h_1 is the length of Pubchem fingerprint and h_2 is the amount of genes in expression profile. The feature selection task for the drug structure or genes on whole genome could be treated as the l_1/l_q -norm regularized multi-class least squares problem and it can be solved with the function mcLeastR in SLEP package [4]. For matrix $F^D \in \mathbb{R}^{M\times h_1}$ and $F^C \in \mathbb{R}^{N\times h_2}$, corresponding matrices $P \in \mathbb{R}^{h_1\times k}$ and $Q \in \mathbb{R}^{h_2\times k}$ will be calculated to represent $U \approx F^D P$ and $V \approx F^C Q$ through the following objective function respectively:

$$\min \frac{1}{2} \| F^D P - U \|_2^2 + \lambda_2 \| P \|_{l_1/l_q}$$

$$\min \frac{1}{2} \| F^C Q - V \|_2^2 + \lambda_2 \| Q \|_{l_1/l_q}$$

Where λ_2 is the l_1/l_q -norm regularization parameter. There are several rows in these two feature matrices that will be zero, indicates that the corresponding features are not important and will not be selected.

REFERENCES

1. Cortes C, Vapnik V. “Support-vector networks”. Machine Learning. 1995; 20:273.
2. Gu Q, Zhou J, Ding C. Collaborative Filtering: Weighted Nonnegative Matrix Factorization Incorporating User and Item Graphs. In Proceedings of the 2010 SIAM International Conference on Data Mining. 199–210.
3. Michael D. Ekstrand, John T. Riedl, Konstan JA: Collaborative Filtering Recommender Systems. Foundations and Trends® in Human-Computer Interaction. 2010; 4:81–173.
4. Jun Liu, Shuiwang Ji, Ye J. SLEP: Sparse Learning with Efficient Projections Arizona State University 2009. http://www. public. asu. edu/ ~jye02/Software/SLEP.

Additional File 2: is a table showing signature genes relevant to drug sensitivity in five different cancer types. breast cancer, hematopoietic and lymphoid cancer, small-cell-lung-cancer, non-small-cell-lung-cancer, and skin cancer.

Additional File 3: is a table listing enriched pathways for the signature genes of the five different cancer types

Breast cancer	
**Term**	***P* Value**
hsa04610:Complement and coagulation cascades	0.047253791
**Hematopoietic and lymphoid cancer**	
**Term**	***P* Value**
hsa04640:Hematopoietic cell lineage	0.004716048
hsa05310:Asthma	0.004989787
hsa04672:Intestinal immune network for IgA production	0.006430767
hsa04630:Jak-STAT signaling pathway	0.012874704
hsa05221:Acute myeloid leukemia	0.012992697
hsa04060:Cytokine-cytokine receptor interaction	0.025147323
hsa05200:Pathways in cancer	0.027689927
hsa04666:Fc gamma R-mediated phagocytosis	0.027741698
hsa05322:Systemic lupus erythematosus	0.033086836
**Non-small cell lung cancer**	
**Term**	***P* Value**
hsa00340:Histidine metabolism	0.029971773
hsa00330:Arginine and proline metabolism	0.035552536
hsa00051:Fructose and mannose metabolism	0.045110355
hsa04512:ECM-receptor interaction	0.04746861
**Small-cell lung cancer**	
None	
**Skin cancer**	
**Term**	***P* Value**
hsa04512:ECM-receptor interaction	1.02E–04
hsa04510:Focal adhesion	0.001149485
hsa05222:Small cell lung cancer	0.004723939
hsa05200:Pathways in cancer	0.026889448

Additional File 4: is a table summarizing synthetic lethality pairs and synthetic dosage lethality pairs composed of the targets of the 94 drugs and the corresponding signature genes

DRUG	Target	Gene_B
BIBW2992	EGFR	AMIGO2
BIBW2992	EGFR	IGFBP3
BIBW2992	EGFR	SFN
BIBW2992	EGFR	TM4SF1
BIBW2992	ERBB2	ERBB2
BIBW2992	ERBB2	PGAP3
Gefitinib	EGFR	AMIGO2
Gefitinib	EGFR	IGFBP3
Gefitinib	EGFR	SFN
Gefitinib	EGFR	TM4SF1
Erlotinib	EGFR	AMIGO2
Erlotinib	EGFR	IGFBP3
Erlotinib	EGFR	SFN
Erlotinib	EGFR	TM4SF1
Lapatinib	EGFR	AMIGO2
Lapatinib	EGFR	IGFBP3
Lapatinib	EGFR	SFN
Lapatinib	EGFR	TM4SF1
Lapatinib	ERBB2	ERBB2
Lapatinib	ERBB2	PGAP3
ZD–6474	EGFR	AMIGO2
ZD–6474	EGFR	IGFBP3
ZD–6474	EGFR	SFN
ZD–6474	EGFR	TM4SF1

Additional File 5: is a table listing drug resistance related genes for each of the 94 drugs

Additional File 6: is a table giving drug-cell line response information for 94 drugs on 608 cancer cell lines

Additional File 7: is a table showing signature genes selected from NSCLC cell line data for mapping to the expression profile of the TCGA NSCLC tumor samples
